# Optical clearing of the mouse skull

**DOI:** 10.1038/s41377-022-00989-0

**Published:** 2022-09-29

**Authors:** Chris Xu

**Affiliations:** School of Applied and Engineering Physics, Cornell University, Ithaca, NY 14853 USA

**Keywords:** Transformation optics, Imaging and sensing

## Abstract

High spatial resolution imaging of the mouse brain through the intact skull is challenging because of the skull-induced aberration and scattering. The research group of Dan Zhu from Huazhong University of Science and Technology has developed a skull-clearing technique that provides a long-term (~ weeks), stable, transparent window for high resolution optical imaging over a large field of view.

Non-invasive imaging of the mouse brain in its native environment is critical to the study of neural network function and disease progression. Current high-resolution mouse brain imaging typically requires either an open-skull craniotomy or thinned-skull preparation^1,2^ because of the strong scattering and aberration induced by the skull bone. Even a thin layer of bone (e.g., 10 s of μm thick) can cause significant degradation of the imaging performance and limit the penetration depth of high-resolution optical imaging. On the other hand, it was shown that open skull surgery can lead to a variety of physiological responses, such as microglial activation, loss of cranial pressure and cerebrospinal fluid, etc. In some cases, the invasiveness of the open-skull procedure may negatively impact the experimental data^3^. In addition, open-skull craniotomy and thinned-skull preparations often significantly restrict the accessible brain areas. Only the regions directly under the skull window can be imaged, which severely limit the ability to investigate animal behaviors that are distributed across multiple, spatially separated regions of the brain. It was shown previously that even simple animal behavior involves multiple brain regions, which are often non-contiguous^4,5^. Long wavelength 3-photon microscopy was shown to enable imaging through the intact mouse skull^6,7^. With the addition of adaptive optics, deep, through-skull 3-photon imaging was possible^8^. Nonetheless, when compared to that of open-skull craniotomy^9,10^, the penetration depth of through-skull 3-photon imaging is significantly reduced. Therefore, high-performance imaging through the intact skull will open new opportunities in brain research.

Skull-clearing is another possible approach for imaging through the intact skull, and researchers have used biocompatible reagents to make the skull itself transparent in the past, with the goal of providing a large window for optical imaging without craniotomy or skull thinning. However, the techniques developed in the past cannot provide a stable window for long term observation or a highly transparent skull.

In the article in eLight^11^, Dan Zhu group from Huazhong University of Science and Technology demonstrated an improved skull-clearing methods, i.e., through-intact-skull (TIS) window, that could keep a large area of the mouse skull transparent for high-resolution optical imaging for weeks, as shown in Fig. 1. A key innovation is using a UV-curable adhesive to stabilize the transparent state of the skull for long term optical imaging. Previous skull optical clearing agents are liquid-based^12–14^. They are unstable on the skull, particularly when the mouse moves, and optical clearing of the skull is needed every time before imaging. The UV-curable adhesive is stable on the skull and unperturbed by animal motion. Furthermore, the UV-curable adhesive has a higher refractive index than the water-soluble reagents used in the past and thus provides better matching of the refractive index to the skull.

**Fig. 1:**
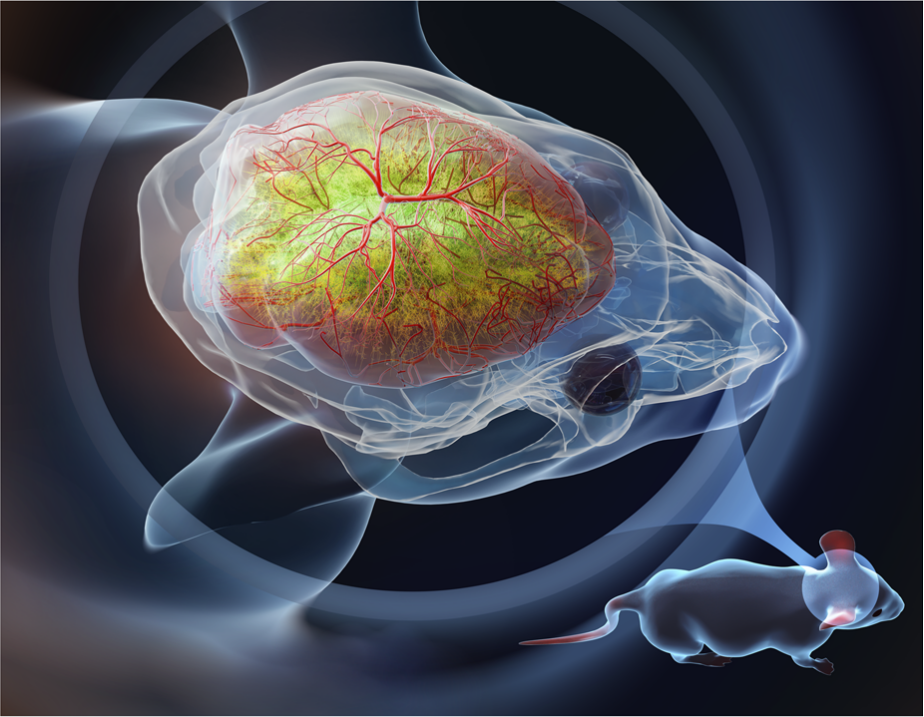
TIS chronic window for imaging the mouse brain structure and function

The TIS window is combined with both 2- and 3-photon microscopy for brain imaging without open-skull craniotomy or thinned-skull preparation. An impressive imaging depth of ~800 μm is realized while maintaining cellular-level spatial resolution. At shallower depth, dendritic spines are resolvable with standard 2-photon microscopy. The optically cleared field-of-view is large (~1 cm), enabling observation of neurons and blood vessels in both hemispheres of the mouse brain. TIS window is compatible with high-resolution neural activity imaging in awake animals over a period of several weeks. A key advantage of TIS window is large-scale, high-resolution imaging with minimum invasiveness to the brain. As a tour de force, TIS window was used to track immune cells over distances of several millimeters, in multiple regions that are millimeters apart from each other and in both hemispheres of the mouse brain, right after acute traumatic brain injury.

The improvement of optical clearing techniques, such as the TIS window, together with the advancement of multiphoton imaging techniques, expands the scope, depth, as well as an available tool kit to push the boundaries of brain research.

## References

[1] Yang G, Pan F, Parkhurst CN, Grutzendler J, Gan W-B (2010). Thinned-skull cranial window technique for long-term imaging of the cortex in live mice. Nat. Protoc..

[2] Drew PJ (2010). Chronic optical access through a polished and reinforced thinned skull. Nat. Methods.

[3] Xu HT, Pan F, Yang G, Gan W-B (2007). The choice of cranial window type for in vivo imaging significantly affects dendritic spine turnover in the cortex. Nat. Neurosci..

[4] Hernandez A (2010). Decoding a perceptual decision process across cortex. Neuron.

[5] Guo Z (2014). Flow of cortical activity underlying a tactile decision in mice. Neuron.

[6] Wang T (2018). Three-photon imaging of mouse brain structure and function through the intact skull. Nat. Methods.

[7] Wang T, Xu C (2020). Three-photon neuronal imaging in deep mouse brain. Optica.

[8] Qin, Z. et al. Deep tissue multi-photon imaging using adaptive optics with direct focus sensing and shaping. *Nat. Biotechnol.*10.1038/s41587-022-01343-w (2022). 10.1038/s41587-022-01343-w 35697805

[9] Ouzounov DG (2017). In vivo three-photon imaging of activity of GCaMP6-labeled neurons deep in intact mouse brain. Nat. Methods.

[10] Liu H (2019). In vivo deep-brain structural and hemodynamic multiphoton microscopy enabled by quantum dots. Nano Lett..

[11] Li D (2022). A Through-Intact-Skull (TIS) chronic window technique for cortical structure and function observation in mice. eLight.

[12] Wang J, Zhang Y, Xu TH, Luo QM, Zhu D (2012). An innovative transparent cranial window based on skull optical clearing. Laser Phys. Lett..

[13] Zhang C (2018). A large, switchable optical clearing skull window for cerebrovascular imaging. Theranostics.

[14] Li D-Y (2020). Visible-near infrared-II skull optical clearing window for in vivo cortical vasculature imaging and targeted manipulation. J. Biophotonics.

